# Metabolic Syndrome and Cardiovascular Risk Factors in Obese Adolescent

**DOI:** 10.3889/oamjms.2016.037

**Published:** 2016-03-02

**Authors:** Manal Mansour, Yasser E. Nassef, Mones Abu Shady, Ali Abdel Aziz, Heba A. El Malt

**Affiliations:** 1 *Child Health Department, National Research Center, Cairo, Egypt*; 2 *Biochemistry Department, National Research Center, Cairo, Egypt*

**Keywords:** cholesterol, cardiovascular diseases, body mass index, Blood glucose level, C-reactive protein

## Abstract

**BACKGROUND:**

Childhood and adolescent obesity is associated with insulin resistance, abnormal glucose metabolism, hypertension, dyslipidemia, inflammation, liver disease, and compromised vascular function. The purpose of this study was to determine the prevalence of cardiovascular risk factor abnormalities and metabolic syndrome in a sample of obese adolescent as prevalence data might be helpful in improving engagement with obesity treatment in future. The high blood lipid levels and obesity are the main risk factors for cardio vascular diseases. Atherosclerotic process begins in childhood.

**AIM:**

This study aimed to investigate the relationship between obesity in adolescent and their blood lipids levels and blood glucose level.

**METHODS:**

This study was conducted with 100 adolescents of both gender age 12-17 years and body mass index (BMI) greater than 95th percentiles and 100 normal adolescents as control group. The blood samples were collected from all adolescents after overnight fasting (10 hours) to analyze blood lipids (Total cholesterol, high density lipoprotein, low density lipoprotein) and hematological profile (Hemoglobin, platelets and red blood cell, C reactive protein and fasting blood glucose.

**RESULTS:**

There were statistical difference between the two groups for red blood cells (P<0.001), Hemoglobin (P < 0.001) and platelets (P = 0.002), CRP (P = 0.02). Positive correlation was found between the two groups as regards total cholesterol (P = 0.0001), P value was positive for HDL (P = 0.005 and Atherogenic index P value was positive (P = 0.002). Positive correlation was found between the two group as regards fasting blood glucose (P = 0.001).

**CONCLUSION:**

Saturated fat was associated with elevated lipid levels in obese children. These results reinforce the importance of healthy dietary habits since child-hood in order to reduce the risks of cardiovascular diseases in adulthood.

## Introduction

Obesity has reached epidemic rate, both in develop and developing countries [1]. It is notably that overweight children and adolescents have higher possibility of becoming obese adults and represent health-related problems early in life including, diabetes, cardiovascular disease (CVD), anddyslipidemia. Dyslipidemia characterized by altered circulating levels of blood lipids and/or lipoproteins concentrations. Alterations in lipid profiles are associated with the development of atherosclerotic plaques which in turn have a relationship with high fat mass, in particular visceral fat [2]. The prevention of cardiovascular diseases must begin decades prior to the onset of symptoms to be effective. There are two types of preventions, the first one is preventing the development of risk factors and the second is managing those children with risk factors [3]. Due to scarcity of dyslipidemia longitudinal studies in childhood and adulthood cardiovascular diseases are needed. Study showed association between body mass index (BMI) in childhood as early as 7-13 years and heart failure in adulthood. Among the risk factors tracking young age, the obesity and cholesterol levels are the strongest ones [4]. Obesity related metabolic syndrome is associated with increase in the levels of a number of markers of inflammation especially CRP. This subclinical or low grade inflammatory state in simple obesity is associated with increased risk for cardiovascular disease and diabetes

The environmental factors, such physical activity and dietary factors can influence the lipid levels and this relationship known also in adults.

Obesity has been described as an important marker of changes in cytokine concentrations and platelets count. Platletes, red blood cells (RBCs) and Hemoglobin are associated with cardio-respiratory conditions, oxidative metabolism, and cardio vascular events in obese children [5].

## Subjects and Method

Written consents were taken from parents and children caregivers to participate in this study. The inclusion criteria were children with high body mass index (BMI). BMI ≥ 95^th^ percentile according to age and gender that should undergo through biochemical tests. The exclusion criteria were overweight and obese children with any endocrine, renal, heart, or liver diseases. The inclusion criteria of children in this study agreed with the recent American Academy of Pediatric recommendation (fasting lipid profile screening for youths with BMI ≥ 95^th^ percentile).

This study was conducted with 100 obese adolescents (40 girls and 60 boys). All adolescents had BMI ≥ 95^th^ percentile, their ages were 12-17 years old and 100 control adolescents with the same age group, all children were free from chronic diseases. Adolescents were recruited from child health department clinic and from private clinic in Giza. Written consents were taken from parent and care givers of the students prior to study.

### Biochemical Investigations

Blood collection was performed 3ml from the cubital viens into a tube containing (EDTA) vacutainer after overnight fasting. RBCs, Hemoglobin and platelets counts were determined by fluorescence flow cytometry (Labquest, Labtest Diagnostica). Total cholesterol (TC), and high density lipoprotein (HDL), low density lipoprotein (LDL) were analyzed using the enzymatic colorimetric methods [6]. LDL was calculated usingFriedevald equation. C reactive protein was evaluated through a quantitative technique, value: 0.1-0.8 mg/dl considered as normal.

Blood glucose was done by clinical chemistry automatic analyser (Dade Behring).

### Clinical assessment

A thorough physical examination was done in all subjects to exclude any significant systemic illness. Blood pressure was measured with a mercury sphygmomanometer after 20min of rest in supine position.

### Anthropometric measurement

Anthropometric measurements were including weight, height, and waist, circumference.

Balanced scale, with the subject standing barefoot and wearing light clothing, weight was recorded in kilograms to the nearest tenth. Height was measured using a rural attached to scale and was recorded to the nearest 0.5 cm. Waist circumference was measured using a flexible, inextensible tap to the nearest 0.5.

### Statistical analysis

It included Student’s (t) test for comparison of mean, simple correlation analysis, and multiple regression using SPSS program of personal computer, all reported p-value were tow tailed, value <0.05 was considered significant.

## Results

Anthropometric measurement and blood pressure of the studied groups are shown in the Table 1. All investigated parameters are significantly different between the control group and obese participants, except age.

**Table 1:** Anthropometric measurement and blood pressure of the studied groups

Parameter	Obese	Control group	P value
Age (Yrs)	11.5 ± 2.28	11.7 ± 0.73	0.404
BMI (Kg/m^2^)	24.4 ± 2.82	17.5 ± 1.65	0.001
WC (cm)	77.7 ± 9.2	60.5 ± 4.52	0.001
Hip C (cm)	86.8 ± 8.67	78.7 ± 5.58	0.001
WHR	0.89 ± 0.79	0.76	0.001
SBP	116 ± 9	109 ± 8	0.001
DBP	72 ± 8	68 ± 6	0.003

**Abbreviations:** (BMI) body mass index, (wc) waist circumference,(HC) hip circumference, (WHC) waist hip circumference,(SBP) systolic blood pressure, (DBP) Diastolic blood pressure.

Comparison of biochemical parameters among two groups are shown in Table 2. All investigated parameters are significantly different between the control group and obese participants.

**Table 2:** Comparison of biochemical parameters among two groups

Parameter	Obese	Control group	P value
TC (mg/dl)	157 ± 32.39	144.4 ± 22.09	0.001
HDL (mg/dl)	36.4 ± 8.26	39.3 ± 6.27	0.005
LDL (mg/dl)	95.4 ± 26.77	88.3 ± 18.45	0.032
AI	2.62 ± 0.91	2.26 ± 0.45	0.002
FBG (mg/dl)	89.8 ± 9.86	81.6 ± 8.71	0.001

AI: atherogenic index; FBG: fasting blood glucose.

Blood indices and C reactive protein in obese and normal adolescent participants are shown in Table 3. All investigated parameters are significantly different between the control group and obese participants.

**Table 3:** Blood indices and C reactive protein in obese and normal adolescent

Variable	Obese	Normal	P value
RBCs (x^12^/L)	5.2 ± 0.4	4.6 ±0.3	<0.001
Hemoglobin (g/L)	14.5 ± 1.1	12.9 ± 0.6	<0.001
Platelets (x10^9^/L)	336.6 ± 64.4	292.7 ± 73.5	0.002
Leukocytes/ul (x^l2^/L)	14100 ± 100	12600 ± 100	0.07
CRP	0.9 ± 0.8	0.79 ± 0.8	0.02

CRP: C- reactive protein.

## Discussion

In the present conditions that obesity is increasing at global level metabolic syndrome will always be in the top of medical problems. Our study is very actual as metabolic syndrome is very important for current medical practice due to a progressive increasing frequency and atherogenic risk [7]. Metabolic syndrome may affect most of the population and it may generate both vascular and metabolic complications [8]. The severity of inflammation in the metabolic syndrome measured by determining C-reactive protein and leukocytes is influenced by the number of criteria that make up metabolic syndrome [9]. Pro-inflammatory mechanisms can be considered as a base of increased cardiovascular risk.

Obesity related metabolic syndrome is associated with increase in the levels of a number of markers of inflammation especially CRP [10]. This subclinical or low grade inflammatory state in simple obesity is associated with increased risk for cardiovascular disease and diabetes [11]. Detection of this systemic inflammation may help to identify children and adolescents at high risk for developing cardiovascular disease and diabetes later in the adulthood. In this study obese adolescent had higher systolic and diastolic blood pressure, P value < 0.001 for systolic blood pressure and P = 0.003 for diastolic pressure. As regards fasting blood glucose p = 0.001 between the two groups. Total cholesterol was higher in obese than normal P value = 0.001, HDL was higher in obese rather than normal P = 0.005 and atherogenic index was higher in obese group than normal P = 0.002.

It has been reported that BMI and waist circumference are strong predicator of central body fatness. The increase of body weight and adiposity, in particular central depots in childhood and adolescents are associated to change in the metabolic profile and cardiovascular problems even early in adult life [12].

The mean systolic and diastolic blood pressures differed significantly amongst the two study groups although none of the subjects were actually hypertensive. These results are consistent with some previous studies [13]. Our observation adds to the hypothesis that inflammatory state that occurs in obesity may contribute to elevation of blood pressure [14]. Elevated blood pressure and in particular the pulse pressure has been associated with an increased risk of cardiovascular disease and the underlying mechanism may be inflammation as demonstrated by elevated CRP levels [15]. Hypertension may also increase cardiovascular risk by causing chronic endothelial injury promoting structural and functional vascular alterations, especially in the microvascular network.

In the present study positive correlation were found between total cholesterol, in obese adolescent and normal (P= 0.04, P=0.03). Atherosclerosis and metabolic syndrome take long time to appear after middle age. This finding were similar to another study were obese adolescents had a significantly more atherogenic lipid profile. Other study stated that having a body mass index outside the normal range significantly worsen risk parameters for cardiovascular diseases in adolescents [16]. Some studies describe non alcoholic fatty liver disease in an early childhood due to dyslipidemia, which is a leading risk factor for cardio-vascular disease [17]. The inclusion criteria of children in this study agreed to the recent American Academy of Pediatric recommendation [10] (fasting lipid profile screening for youths with BMI ≥ 85th percentile on the CDC growth charts [18]). Due to the scarcity of dyslipidemia longitudinal studies in childhood and adulthood cardiovascular diseases, there are several speculations some studies found that obese children aged 7 and 13 year and the ratio of heart failures.

Furthermore, there is also an epidemiologic cardiovascular risk in children with dyslipidemia and tracking indicates that children maintain their percentile ranking over the time [19]. Among the risk factors tracking young age through adulthood, the obesity and cholesterol levels are the strongest ones [20]. The environmental factors, such physical activity and dietary factors can influence the lipids levels and this relationship is known in adults. However, there are few studies that evaluated the influence of dietary factors in childhood [21].

Growth acceleration in early life may be a predictor for obesity later on. BMI is an important parameter to determine obesity. There were very high positive correlation between anthropometric parameters in the two group (p = 0.00 for height, P = 0.03 for weight, P = 0.001 for BMI, and P = 0.001 for waist circumference), hip circumference (HC) P = 0.001.

Waist circumference is indicator for central obesity and should be added to every measurement of obesity. Although imaging techniques can determine total body fat and its distribution reliably, anthropometric measurements remain important in clinical practice. Body mass index and waist circumference are reliable, save and non expensive method in clinical practice [22].

Childhood obesity become a serious public health problem, nutritional therapy plays an important role in its prevention and treatment. Healthy life style measures should be encouraged in this group of adolescents to prevent metabolic syndrome and cardiovascular complications in adulthood.

In conclusion, saturated fat was positively associated with elevated lipid levels in obese schoolchildren. This study suggests that number of cardiometabolic risk factors and metabolic syndrome are prevalent in obese adolescents. This observation might provide impetus to future strategies to treat pediatric obesity and to prevent or delay the appearance of cardiovascular disease and diabetes mellitus in future adult generation. These results reinforce the importance of healthy dietary habits since child-hood. The observation might also be used to encourage greater engagement among families.
